# Free androgen index (FAI)’s relations with oxidative stress and insulin resistance in polycystic ovary syndrome

**DOI:** 10.1038/s41598-023-31406-0

**Published:** 2023-03-29

**Authors:** Leili Rahmatnezhad, Lida Moghaddam-Banaem, Tahereh Behrouzi Lak, Afshin Shiva, Javad Rasuli

**Affiliations:** 1 Department of Reproductive Health and Midwifery, Faculty of Medical Sciences, Tarbiat Modares University, Tehran, Iran; 2 Department of Infertility, Urmia University of Medical Sciences, Urmia, Iran; 3 Experimental and Applied Pharmaceutical Sciences Research Center, Urmia University of Medical Sciences, Urmia, Iran; 4 Department of Epidemiology and Biostatistics, Faculty of Medicine, Urmia University of Medical Sciences, Urmia, Iran

**Keywords:** Biomarkers, Endocrinology, Health care, Medical research

## Abstract

This study aimed to determine the levels of the free androgen index (FAI) and its association with oxidative stress and insulin resistance (IR) in patients with polycystic ovary syndrome (PCOS). This cross-sectional study was performed on 160 women aged 18–45 years, visiting gynecology clinics of Urmia in northwestern Iran during 2020–2021 who were diagnosed with PCOS and exhibited one of the four phenotypes of PCOS. All the participants underwent clinical examinations, paraclinical tests, and ultrasounds. FAI cut-off point was considered to be 5%. The significance level was set at < 0.05. Among the 160 participants, the prevalence of the four phenotypes was as follows: phenotype A: 51.9%, phenotype B: 23.1%, phenotype C: 13.1%, and phenotype D: 11.9%. High FAI was detected in 30 participants (18.75%). Additionally, It was found that phenotype C had the highest FAI levels among the PCOS phenotypes, with a significant difference between phenotypes A and C (*p* value = 0.03). IR was observed in 119 (74.4%) of the participants, and the median (interquartile range: IQR) of malondialdehyde (MDA) levels among the participants was 0.64 (0.86) μM/L. In linear regression, the PCOS phenotype (standard beta = 0.198, *p*-value = 0.008), follicle-stimulating hormone (FSH) levels (standard beta = 0.213, *p*-value = 0.004), and MDA levels (standard beta = 0.266, *p*-value < 0.001) were significantly related to the FAI level, but the homeostatic model assessment for insulin resistance (HOMA-IR) was not statistically associated with FAI. Thus, in this study, PCOS phenotypes and MDA levels (an indicator of stress oxidative) were significantly related to FAI, but HOMA-IR (the indicator of IR) was not associated with it.

## Introduction

Polycystic ovary syndrome (PCOS) is an endocrine metabolic disorder, which afflicts 7% to 10% of women of reproductive age^1^. It is a multisystem endocrine disorder, which is characterized by clinical and biochemical abnormalities such as menstrual irregularities, hyperandrogenism, infertility, hyperinsulinemia, and multiple ovarian cysts^2^. According to the Rotterdam criteria, PCOS is defined by the presence of two of the following criteria (by ruling out other etiologies): reduced or absent ovulation, clinical or biochemical hyperandrogenism, and polycystic ovaries^3^.

According to the Rotterdam criteria for PCOS, there are four different phenotypes for this syndrome: (A): oligomenorrhoea + polycystic ovaries (PCO) + hyperandrogenism, (B): oligomenorrhoea + hyperandrogenism, (C): hyperandrogenism + PCO, and (D): oligomenorrhea + PCO.

High concentrations of testosterone, total cholesterol, and LDL cholesterol in phenotype A increase the risk of cardiovascular diseases, type 2 diabetes, or metabolic syndrome^4^.

This disorder is initially characterized by irregular menstrual cycles with no ovulation^5^. The absence of ovulation is one of the main characteristics of PCOS, and its spectrum ranges from normal ovulation to long-term ablation^6^. After oligomenorrhea, hirsutism is the second most common symptom of PCOS^7^.

Hyperandrogenism is also a proven factor in the development of this syndrome with a prevalence of 60–80%, and insulin resistance (IR) is another known factor contributing to the development of PCOS, which is observed in 50–80% of patients^8^. Hyperandrogenism in PCOS leads to hirsutism, acne, and alopecia. Androgens may also inhibit follicular growth and thus prevent ovulation7.

IR and the resulting compensatory hyperinsulinism are associated with hyperandrogenemia in several ways. Insulin increases androgen synthesis from theca cells, either directly or by increasing the theca cell response to the circulating luteinizing hormone (LH)^9^.

Oxidative stress, which is defined as an imbalance resulting from the excessive formation of oxidants in the presence of limited antioxidant defenses, is actively involved in the etiology of the syndrome in addition to hormonal disturbances, insulin signaling defects, and adipose tissue dysfunction^10^.

It has been shown that oxidative stress affects the ovarian phases and leads to hormonal disorders that disturb the hormonal conditions, causing the disorder to become more severe^11^.

Malondialdehyde (MDA) is a marker of chronic oxidative stress^12^. Biomarkers of oxidative stress have the potential to be used in the evaluation of the risk of oxidative damage and related diseases^13,14^. Among the products of lipid peroxidation are malondialdehyde (MDA) and hydroxyl radicals, which accumulate from damaged intracellular and cell wall polyunsaturated fatty acids, along with an increase in ROS. It is therefore likely that serum MDA levels could serve as a measure of lipid peroxidation as well as damage to the membrane and DNA of cells^15^. Thus, MDA can be employed as an indicator in assessing the effectiveness of antioxidant therapy^12^.

Functional ovarian hyperandrogenism is a type of PCOS characterized by increased circulating levels of ovarian-derived androgens. IR is the most common etiologic factor in women with functional ovarian hyperandrogenism. It increases oxidative stress and worsens antioxidant status. Oxidative stress is directly related to IR and testosterone levels, thereby contributing to endocrine and biochemical changes in women with functional ovarian hyperandrogenism^16^.

Given the importance of FAI in diagnosing functional ovarian hyperandrogenism as a type of PCOS and its association with increased oxidative stress and other individual characteristics and considering that no study has been conducted in this field on different PCOS phenotypes, the present research aimed to investigate the prevalence of high FAI levels in PCOS and its association with oxidative stress and insulin resistance in PCOS patients.

## Results

Out of 168 women with PCOS who met the inclusion criteria for this study, 160 gave their consent to participate in the study. The prevalence of the four phenotypes and characteristics of the participants are shown in Table 1.

**Table 1:** Demographic, clinical, and paraclinical characteristics of the participants (n = 160).

Characteristic (Qualitative)	Grouping	N (%)	Characteristic (Quantitative)	Median (IQR)
Marital status	Single	96 (60)	Age (years)	24 (7)
	Married	64 (40)	Height (cm)	165 (6)
			Weight (kg)	66.5 (14.75)
Job	Employed	14 (8.8)	BMI (kg/m2)	24.61 (5.38)
	Housewife	57 (35.6)	Age at menarche (years)	12 (1)
	University student	51 (31.9)	FSH (IU/L)	2.73 (1.47)
	Highschool student	19 (11.9)	Total testosterone (ng/ml)	0.65 (0.58)
	Self-employment	19 (11.9)	SHBG (nmol/L)	31.3 (14.58)
Education	Illiterate	5 (3.1)	LH (IU/L)	7.45 (5.9)
	Under diploma	33 (20.6)	MDA (μM/L)	0.64 (0.86)
	Diploma	29 (18.1%)	FBS (mg/dl)	80 (2)
	College education	93 (58.1%)	FI (μU/dl)	19.65 (16.37)
			HOMA	3.88 (3.26)
Economic status	Poor (expenditure more than income)	44 (27.5%)	Hb (g/dl)	12.30 (0.30)
	Good (expenditure less than and equal to income)	116 (72.5%)	TSH (mU/L)	3.20 (1.04)
Physical activity	No (< 90 min per week)	66 (41.3%)	T4 (nmol/L)	1.07 (24)
	Yes (> = 90 min per week)	94 (58.8%)	T3 (nmol/L)	1.20 (0.85)
PCOS Phenotype	A	83 (51.9%)	PLT (N/mm3)	209.50 (66.75)
	B	37 (23.1%)	FAI	2.04 (2.48)
	C	21 (13.1%)	WHR	0.81 (.02)
	D	19 (11.9%)		
FAI	Low (< 5%) (n:130 )	130 (81.25%)		
	High (> = 5%) (n:30 )	30 (18.75%)		
Insulin Resistance (IR)	No (HOMA < 2.5)	41 (25.6%)		
	Yes (HOMA > = 2.5)	119 (74.4%)		
BMI	Normal (< 25)	85 (53.1%)		
	Overweight/obese (> = 25)	75 (46.9%)		
Ovarian cysts	N < 2	7 (4.4%)		
	N > = 2	153 (95.6%)		

BMI: Body mass index, FSH: Follicle Stimulating Hormone, SHBG: Sex Hormone Binding Globulin, LH: Luteinizing Hormone, MDA: Malonaldehyde, FBS: Fasting Blood Sugar, FI: Fasting Insulin, HOMA-IR: Homeostasis Model Assessment of Insulin Resistance, Hb: Hemoglobin, TSH: Thyroid Stimulating Hormone, T4: Thyroxin, T3: Triiodothyronine, PLT: Platelets, FAI: Free Androgen Index, WHR: Waist-to-Hip Ratio.

According to Rotterdam criteria, out of the 160 women studied, 83 (51.9%) had phenotype A, which was the most prevalent phenotype compared to the others. Considering the FAI cut-off point of 5%, 30 patients (18.75%) had High FAI. In addition, 85 patients (53.1%) had normal body mass index (BMI). The median and interquartile range (IQR) of FAI in the participants was 2.04 (2.48) the median and IQR of MDA levels in the participants were 0.64 (0.86). The relationships between FAI and demographic, clinical, and paraclinical characteristics of the participants are provided in Table 2.

**Table 2:** The relationships between FAI and demographic, clinical, and paraclinical characteristics of the participants (n = 160).

Parameter (quantitative)	FAI		
	Correlation coefficient (r)	*P*-value (Spearman's test)	
Age (years)	0.03	0.7	
Height(cm)	0.02	0.76	
Weight(kg)	0.02	0.78	
Age at menarche (years)	**0.16**	**0.03**	
FSH (IU/L)	0.10	0.18	
LH (IU/L)	0.10	0.2	
MDA (μM/L)	**0.30**	** < 0.001**	
Hb (g/dl)	-0.03	0.64	
TSH (mU/L)	0.04	0.57	
PLT (N/mm3)	0.01	0.88	
BMI (kg/m2)	0.006	0.93	
HOMA	**0.17**	**0.02**	
WHR	0.05	0.5	

Parameter (Qualitative)	Grouping	FAI Median( IQR)	*P*-value
Education	Illiterate (n:5)	4.17 (4.07)	0.17 ■
	Under diploma/Diploma (n:62 )	2.04 (2.17)	
	College education (n:93)	1.96 (2.61)	
Number of ovarian cysts	< 2 (n:7)	2.87 (4.31)	0.63⬜
	> = 2 (n:153)	2.03 (2.3)	
Job	Housewife (n:57)	2.14 (2.73)	0.79⬜
	Employed (n:103)	2.03 (2.4)	
PCOS phenotype^△^	A (n:83)	1.75 (1.99)	△**0.03 ■**
	B (n:37)	2.46 (3.67)	
	C (n:21)	3.05 (4.99)	
	D (n:19)	2.03 (4.24)	
Marital status	Single (n:96)	1.94 (2.5)	0.27⬜
	Married (n:64)	2.31 (2.62)	
Physical activity	No (< 90 min per week) (n:66 )	2.04 (2.22)	0.32⬜
	Yes (> = 90 min per week) (n:94 )	2 (2.75)	
Economic group	Poor (expenditure more than income) (n:44 )	1.96 (2.02)	0.71⬜
	Good (expenditure less than income) (n:116 )	2.07 (2.81)	
BMI group (kg/m2)	Normal (< 25) (n:85 )	2.09 (2.42)	0.86⬜
	Overweight/obese (> = 25) (n:75 )	2.03 (2.58)	

FSH: Follicle Stimulating Hormone, LH: Luteinizing Hormone, MDA: Malonaldehyde, Hb: Hemoglobin, TSH: Thyroid Stimulating Hormone, PLT: Platelets, BMI: Body Mass Index, HOMA-IR: Homeostasis Model Assessment of Insulin Resistance, WHR: Waist-to-Hip Ratio.

⬜ Mann–Whitney U Test, **■** Kruskal–Wallis test.

△ Pairwise comparisons were conducted by the Bonferroni correction for multiple tests. Significant results were: Phenotypes A & C: *P* – value = 0.007.

Significant values are in bold.

Regarding the relationship between FAI and demographic, clinical, and paraclinical variables in the participants, the following parameters were significantly associated with FAI: age at menarche (r = 0.16), MDA level (r = 0.30), and HOMA-IR (homeostatic model assessment for insulin resistance) (r = 0.17) (Table 2). It was found that phenotype C had the highest FAI levels among the PCOS phenotypes, with a significant difference between phenotypes A and C. The relationship between the FAI groups and the demographic, clinical, and paraclinical characteristics of the participants are shown in Table 3.

**Table 3:** The relationship between high FAI (cut-off point 5%) and the demographic, clinical, and paraclinical characteristics of the participants (n = 160).

Parameter (Quantitative)	High FAI (n = 30) Median (IQR)	Normal FAI (n = 130) Median (IQR)	*P*-value (Mann–Whitney Test)	
Age (years)	23.5 (7.5)	24 (7)	0.2	
Height(cm)	165 (8)	165 (6)	0.91	
Weight(kg)	64.5 (15.5)	67 (15.75)	0.74	
age at menarche (years)	12 (0)	12 (1)	**0.03**	
FSH (IU/L)	3.1 (3.48)	2.6 (1.3)	**0.01**	
LH (IU/L)	8.95 (9.7)	7.2 (5.36)	0.13	
MDA (μM/L)	0.6 (1.25)	0.68 (0.89)	0.35	
Hb (g/dl)	12.1 (0.73)	12.3 (0.6)	0.72	
TSH (mU/L)	3.2 (0.85)	3.16 (1.08)	0.46	
T4 (nmol/L)	1.08 (0.23)	1.06 (0.3)	0.52	
PLT (N/mm3)	198 (45)	216.5 (68)	0.52	
BMI (kg/m2)	24.50 (6.01)	24.61 (5.24)	0.82	
HOMA (nmol/L)	4.82 (2.97)	3.74 (3.29)	0.2	
WHR	0.81 (0.03)	0.81 (0.02)	0.67	

Parameter (Qualitative)	Grouping	High FAI (n = 30) N (%)	Normal FAI (n = 130) N (%)	*P*-value (Chi-Square Tests)
Education	Illiterate	2 (40)	3 (60)	0.3
	Under diploma and Diploma	9 (14.5)	53 (85.5)	
	college education	19 (20.4)	74 (79.6)	
Number of ovarian cysts group	N < 2	2 (28.6)	5 (71.4)	0.61
	N > = 2	28 (18.3)	125 (81.7)	
PCOS phenotype	A	9 (10.8)	74 (89.2)	0.05
	B	9 (24.3)	28 (75.7)	
	C	7 (33.3)	14 (66.7)	
	D	5 (26.3)	14 (73.7)	
Marital status	Single	15 (15.6))	81 (84.4)	0.22
	Married	15 (23.4)	49 (76.6)	
Exercise	No (< 90 min per week)	15 (22.7)	51 (77.3)	0.3
	Yes (> = 90 min per week)	15 (16)	79 (84)	
Economic status	Week (expenditure more than income)	8 (18.2)	36 (81.8)	1
	Good (expenditure less than income)	22 (19)	94 (81)	
Job	Housewife	12 (21.1)	45 (78.9)	0.67
	Employed	18 (17.5)	85 (82.5)	
Hirsutism	Yes	127 (82.5)	27 (17.5)	0.08
	No	3 (50)	3 (50)	

FSH: Follicle Stimulating Hormone, LH: Luteinizing Hormone, MDA: Malonaldehyde, Hb: Hemoglobin,TSH: Thyroid Stimulating Hormone, T4: Thyroxin, PLT: Platelets, BMI: Body Mass Index, HOMA-IR: Homeostasis Model Assessment of Insulin Resistance, WHR: Waist-to-Hip Ratio.

Significant values are in bold.

Age at menarche and follicle-stimulating hormone (FSH) were significantly associated with high FAI (cut-off point 5%). The highest FAI rate was observed in phenotype C patients (33.3%). Linear regression analyses were used to control confounding factors as shown in Table 4.

**Table 4:** Linear regression analyses to assess the factors effective on FAI in the study participants (n = 160).

Variable	Standardized coefficients beta	*P*-value
PCOS phenotype	0.198	**0.008**
FSH (IU/L)	0.213	**0.004**
MDA (μM/L)	0.266	** < 0.001**
HOMA-IR	0.139	0.06

FSH: Follicle Stimulating Hormone, MDA: Malonaldehyde, HOMA-IR: Homeostasis Model Assessment of Insulin.

Significant values are in bold.

In linear regression, FAI was entered as the independent variable and its possible effective factors as independent variables. The results showed that PCOS phenotypes, FSH, and MDA levels were significantly related to FAI.

## Discussion

The present study was conducted on 160 women with PCOS in Urmia Province in the northwest of Iran, in order to determine the FAI level and its association with oxidative stress and IR in PCOS patients.

The most prevalent PCOS phenotype in this study was phenotype A (51.9%), which was consistent with the results of the study by Vaggopoulos et al. They showed that the prevalence of phenotype A was higher than the other phenotypes^17^. However, the study by Alawia et al. reported different findings from our study. They found that the prevalence of phenotype D was higher than the other phenotypes^18^, which could be due to genetic factors, lifestyle, eating habits, and differences in the number of participants.

In the present study, considering a cut-off point of 5% for FAI, 30 participants (18.75%) had high FAI. According to the results of the present study, the rate of high FAI among different phenotypes of PCOS was as follows: phenotype A (10.8%), phenotype B (24.3%), phenotype C (33.3%), and phenotype D (26.3%), with phenotype C exhibiting the highest rate of high FAI. Moreover, the FAI level was significantly different between the four phenotype groups in our study, and the highest level was observed among phenotype C patients. This finding is inconsistent with the results of Głuszak's study. Their study indicated elevated levels of total testosterone, androstenedione, and significantly higher levels of total cholesterol and LDL cholesterol in the phenotype A group^4^, which could be due to differences in the participants' characteristics and lifestyles.

According to the results, there was a statistically significant relationship between FAI and MDA, which was in line with the results of the study by Desai et al.^19^. According to their findings, IR, hyperandrogenism, dyslipidemia, and obesity associated with PCOS increased MDA levels while decreasing antioxidant enzyme levels. According to their research, oxidative stress leads to cell damage and activates the transcription of pro-inflammatory cytokines such as tumor necrosis factor-alpha, which is a known mediator of IR. This pro-inflammatory state may also contribute to IR and hyperandrogenism^19^.

Moreover, the results of our study were in line with those of the study by Yuan et al. which was conducted in order to investigate the relationship between sex hormone binding globulin (SHBG) and oxidized low-density lipoprotein (ox-LDL), total oxidant status, total antioxidant capacity, oxidative stress index, and MDA and to evaluate the effect of oxidative stress on SHBG expression. Their findings showed that oxidative stress inhibits the expression and secretion of SHBG in laboratory conditions and may be an important factor in increasing the incidence of hyperandrogenemia in PCOS^20^.

We also found a statistically significant direct correlation between FAI and HOMA-IR, which was in line with the results of the study by Garzia et al. They stated that the insulin-induced increase in androgens is primarily due to the direct effect on the steroidogenesis of the ovarian theca cells as well as the inhibitory effect on the production of insulin-like growth factor 1 (IGF-1) binding protein by the liver^21^.

In this study, a statistically significant relationship was observed between MDA and HOMA. The findings of our research were in line with those of the study by Uçkan et al., which aimed to investigate the relationship between the oxidant-antioxidant status, endothelial dysfunction, lipid metabolism, and the risk of metabolic syndrome in women with PCOS. They reported a positive correlation between MDA, BMI, and HOMA-IR in the PCOS patient group^22^. Furthermore, our findings were in line with those of the study by Fatima et al., which was conducted in order to evaluate the relationship between PCOS and oxidative stress as well as the relationship between oxidative stress biomarkers and insulin parameters. The results of their study showed a positive correlation between oxidative stress and insulin parameters in PCOS^23^.

There was a statistically significant relationship between FAI and age at menarche, which was in agreement with the results of the study by Asanidze et al. They reported that around 50% of adolescents with PCOS (according to Rotterdam and NIH criteria) have biochemical hyperandrogenism, but the cut-off values were not reported in the study^24^. Our findings were also consistent with those of the research by Valeria Calcaterra et al. which reported the earlier onset of menstruation in adolescents with PCOS^9^. In our work, there was no link between hirsutism and the level of FAI, which contradicted the findings of the study by Chanukvadze et al. In their study, which was conducted to investigate the relationship between clinical symptoms and biochemical markers of hyperandrogenism, they reported a positive correlation between the hirsutism score and FAI and a negative correlation between the hirsutism score and SHBG^25^, which could be due to genetic factors, lifestyle, eating habits, and differences in the number of participants.

Multivariate analysis (linear regression) was used to assess the predictive ability of independent variables and to adjust and control the effects of confounding variables. In linear regression, FAI was entered as the independent variable and its possible effective factors as independent variables. The results showed that the phenotype of PCOS, FSH, MDA, and HOMA-IR levels were significantly associated with FAI. According to these results, PCOS phenotype C and high FSH and MDA levels led to an increase in FAI, which should be taken into account in PCOS management and treatment programs.

## Conclusion

The prevalence of PCOS phenotype A was higher than the other phenotypes in our study. The four PCOS phenotypes were significantly different in terms of FAI, and the highest rate of FAI was observed in phenotype C. PCOS phenotypes, FSH, HOMA-IR, MDA, and age at menarche were related to FAI. Therefore, it seems necessary to pay attention to FSH, HOMA-IR, MDA levels, and PCOS phenotypes when diagnosing PCOS for better management of androgens in PCOS patients and the other complications of this disorder; however, further research is suggested.

## Methods

### Ethical approval

The ethics committee of Tarbiat Modares University approved all the procedures of this study (ethical approval no: IR.TMU.REC.1397.235). Moreover, all the methods were performed in accordance with the relevant guidelines and regulations. This cross-sectional study was carried out from November 2020 to June 2021 on 160 women aged 18–45 years who had a PCOS diagnosis and visited the obstetrics and gynecology clinics of Urmia City (a city with a population of 736,224, according to the census of 2016, located in northwestern Iran).

The sampling method used in this study was convenient sampling. All eligible women who met the inclusion criteria were enrolled in the study until the sample size was reached. Inclusion criteria were 18 to 45 years of age; diagnosis of PCOS by a gynecologist (based on clinical, laboratory, and imaging findings); no current pregnancy; not receiving infertility treatment, hormonal medications, or any medications other than over the counter (OTC) painkillers in the last three months; interval of more than four years from the onset of menarche; and the absence of severe underlying diseases such as malignancy and thalassemia affecting menstrual cycles, known endocrinopathies such as Cushing's syndrome, untreated thyroid disorders, and other similar conditions.

### Study procedures

The purpose and protocol of the study were explained and informed consent was obtained from all the participants before enrolment. A researcher-made questionnaire of demographic/reproductive/medical characteristics was completed by the researcher for all the patients. The questionnaire included age, level of education, occupation, marital status, number of pregnancies, number of abortions, number of children, age at menarche, economic status (poor, average, or wealthy as stated by the participant), physical activity (regular exercises or inactive as stated by the participant), diet (the common diet or a specific diet such as vegetarianism), and history of illnesses. After entering the study, relevant clinical examinations, paraclinical tests, and ultrasounds were performed for all the participants as follows:

Anthropometric measurements were performed using standard protocols and calibrated instruments. Height without shoes was measured with a standard tape attached to the wall. Weight was measured with light clothes and without shoes using a Seka 755 scale with an accuracy of 500 g. BMI was calculated using the following formula: weight (kg)/height^2^ (m). Waist circumference was measured with a standard measuring tape parallel to the umbilicus, and hip circumference was measured with a measuring tape at the largest diameter of the hip area. The waist-to-hip ratio (WHR) was measured as the waist/hip circumference.

Clinical signs of hyperandrogenism (acne, oily skin, and hirsutism) were assessed by observation and physical examination. All clinical findings were evaluated by a gynecologist. The diagnosis of hirsutism was based on taking a history and performing a clinical examination with the modified Freeman-Galloway rating score, which examines coarse terminal hairs in nine areas of the body including the upper lip, chin, chest, upper and lower abdomen, arms, and thighs. The severity of hirsutism in each section was scored from 0 to 4^26^. In the summation of scores, women with a level ≥ 4 were considered as hirsute^27^.

Venous blood samples of the participants were collected in fasting conditions (after fasting for 10–12 h) to measure the following indices: Fasting blood sugar (FBS), fasting insulin levels (FIL), total serum testosterone, thyroid stimulating hormone (TSH), T4 (thyroxine), T3 (triiodothyronine), MDA, SHBG, FSH, LH, hemoglobin (Hb), and platelets (PLT).

The serum levels of FSH, LH, total testosterone, insulin, and SHBG were measured using the enzyme-linked immunosorbent assay (ELISA) (Demeditec Diagnostics GmbH, German). The serum levels of FBS were measured using electro-chemical luminescent technique kits (E-411, Roche Company Germany, immunoassay technique). MDA levels were measured using thiobarbituric acid (TBA) with a TBARS kit (KA1381) (Abnova, Taiwan).

### Study variables

#### Hyperandrogenism

Hyperandrogenism was defined based on serum levels of male hormones (total serum testosterone, SHBG, FAI) and clinical signs (acne, oily skin, hirsutism, and male pattern hair loss). FAI was calculated as (total testosterone)/SHBG × 100^28^, and the cut-off point of FAI was set at 5% so that the values above 5% were considered high FAI^29^.

#### PCOS phenotypes

These phenotypes were identified in the study participants based on history, clinical examination, and paraclinical tests using hyperandrogenism (H), ovulatory dysfunction (OD), and PCO as follows: Phenotype A: OD + PCO + H; phenotype B: OD + H; phenotype C: H + PCO; phenotype D: OD + PCO.

#### IR

IR was assessed using HOMA-IR, which was calculated as follows: fasting insulin (mg/dL) × fasting blood glucose/405 (μu/mL)^30^. HOMA-IR (the cut-off value for IR) ≥ 2.5 was considered an indicator of IR according to the previous studies^1,31–34^.

#### BMI

It was calculated using the following formula: weight (kg)/[height (m)]^2^^35^.

#### PCO

PCO was defined as finding 10 or more immature follicles in each ovary and/or an ovarian volume of more than 10 cm^3^ in ultrasound^36^.

#### Menstrual disorders/OD

These disorders included amenorrhea, oligomenorrhea, hypomenorrhea, hypermenorrhea, and irregular menstrual intervals and were defined based on the participants' history. Menstrual disorders were diagnosed as oligomenorrhea when menstrual cycles lasted more than 35 days or occurred less than nine times a year.

### Data management and analysis

Data were entered into the computer and analyzed by IBM® SPSS® Software version 26. The statistical significance level was set at < 0.05. Qualitative variables were compared by the K^2^ test. Quantitative variables in two groups were analyzed by the Mann–Whitney U test due to the non-normal distribution of variables. Quantitative variables in more than two groups were analyzed by the Kruskal–Wallis test due to the non-normal distribution of variables. Linear regression analysis was used to determine effective factors on FAI.

## Data Availability

The datasets that support the findings of the current study are available from the corresponding author upon reasonable request.
